# Empowering health care staff to address health inequalities: A policy-focused evidence brief

**DOI:** 10.1016/j.puhip.2026.100774

**Published:** 2026-03-20

**Authors:** Danielle Lamb, Sashika Harasgama, Amy Dehn Lunn, Camille Gajria, Helena Painter, Helen Pearce, Alice Vodden, Adnaan Ghanchi, John Ford

**Affiliations:** a NIHR Applied Research Collaboration, UCL, UK; b Wolfson Institute for Population Health, Queen Mary University of London, UK; c Institute of Health Sciences Education, Queen Mary University of London, UK; d University College London Hospitals NHS Foundation Trust, UK; e Department of Public Health and Primary Care, University of Cambridge, UK

**Keywords:** Health inequalities, Workforce empowerment, Cultural competency

## Abstract

**The policy challenge:**

Leveraging the health care workforce to take action on health and care inequalities has significant potential, with the NHS in England employing 1.5 million people. Minority ethnic groups are over-represented in the NHS workforce, but underrepresented in senior roles, with high discrimination rates and limited career progression. Chronic workforce shortages exacerbate these issues, increasing workloads and reducing morale. Additionally, inequitable staff distribution, favouring affluent areas over deprived ones, hinders progress in disadvantaged communities. Systemic changes are needed to promote equity in the workforce and close health inequalities gaps. Here, we review the policy-focused evidence that explores how to empower healthcare staff to address inequalities.

**Key evidence to inform policy:**

We identified four key themes of how staff can be empowered to address health and care inequalities: 1) empowering through skills, 2) empowering through resources, 3) empowering through workforce structure, and 4) empowering through staff champions. There is evidence that training in cultural competency, anti-racism, anti-stigma, advocacy, health literacy, and game-based training can empower staff. However, these interventions require adequate time and should be complimented by innovative care models, such as trauma-informed care. Furthermore, the workforce should be diverse at all levels with enhanced professional development for employees from underrepresented backgrounds. Staff champion programmes can promote workforce equality, fostering collaboration and innovation.

**Further considerations and implications:**

Research on staff empowerment to tackle inequalities is limited and often focuses on staff rather than patient outcomes. There are also challenges in measuring cultural competence and comparing studies. Key training areas include cultural competency (ethnicity, LGBTQ+, and mental health), anti-stigma, anti-racism, advocacy, and low health literacy perspectives. Organisations should provide culturally competent resources, promote trauma-informed care, and ensure staff have sufficient time to support patients with complex needs and equity-focused improvements.

## Highlights

• This rapid evidence review identified key themes for staff empowerment in addressing health inequalities.
• Training in cultural competency, anti-racism/stigma, advocacy, and health literacy enhances provision of equitable care.
• Workforce diversity, CPD, and equity-focused leadership programs improve retention/career progression for minority groups.
• Time allocation, culturally competent resources, and TIC are critical to ensure staff can effectively reduce inequalities.

## 1 Current policy challenges

**Fig. 1:**
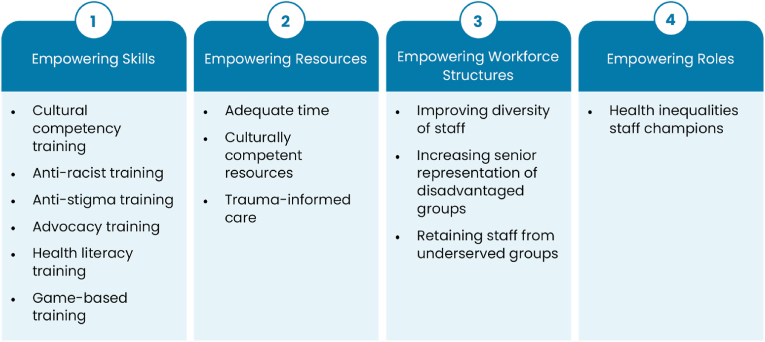
Overview of staff empowerment evidence.

Health inequalities persist as a significant challenge globally, disproportionately affecting marginalised and socioeconomically disadvantaged populations. Given the extensive healthcare systems that exist in many countries, mobilizing current and future healthcare staff, both clinical and non-clinical, to take action to reduce health and care inequalities offers huge potential. For example, in England, the NHS employs 1.5 million people (approximately 1 in 23 of all workers) [1,2], making it the fifth-largest employer globally, with a more diverse workforce than the majority of organisations in the UK [3] (see Fig. 1).

However, diversity within the NHS workforce does not equate to equity in opportunities or experiences for staff themselves. While 25% of NHS staff belong to Asian, Black, or minority ethnic groups—double the proportion within the general population—representation varies across professional roles [4]. For instance, 39% of nurses are from minority ethnic backgrounds compared to only 7% of ambulance staff [4]. Moreover, these staff members experience disproportionately high levels of workplace discrimination; 16.6% reported experiencing discrimination from colleagues compared to 6.7% of their white counterparts [3].

In the UK, the 2023 Workforce Race Equality Standard (WRES) survey further highlights disparities in professional development. Only 39.3% of Black staff felt they had equal opportunities for career progression. While minority representation in senior roles has improved—16% of board members were from minority groups in 2023, a notable increase from 7.4% in 2018—much work remains to ensure equity at all levels [3].

Chronic workforce shortages compound these challenges, with over 107,000 positions unfilled as of September 2024, representing 7.3% of roles across secondary and community care [5]. These vacancies intensify staff workloads, leading to burnout, low morale, and poor retention rates.

The distribution of staff is also inequitable. General practices in affluent areas benefit from more GPs per capita compared to those in deprived regions, a disparity that has worsened over the past eight years [6,7]. This inequity undermines efforts to improve health outcomes in socioeconomically disadvantaged areas.

The aim of this evidence review is to explore what works to empower health care staff to address health and care inequalities. We use ‘empower’ in this context to mean giving staff confidence and autonomy to address these inequalities.

## 2 Approach to collating evidence

To develop policy-relevant recommendations, we undertook a rapid review of the evidence. To navigate a breadth of disparate literature, we prioritised the most robust and relevant studies using a combination of searches strategies. Our aim was to gather sufficient quality and quantity of evidence to inform policy, rather than review all the evidence pertaining to a broad policy area. We undertook a MEDLINE search of reviews published since 2013 that looked at health care workforce interventions relating to disadvantaged groups (based on existing search terms). We focused on a population including healthcare staff of any type, who were being trained, using, or delivering interventions aimed at addressing health inequalities. We only included reviews, which could be narrative, systematic, meta-analyses, or meta-syntheses. We included peer-reviewed studies of all methodological types from all countries, as well as grey literature. We also used the Health Equity Evidence Centre living evidence maps [8,9] and snowballing to identify additional relevant studies. We prioritised 42 relevant studies, 28 of which were judged to be major contributors to policy decision making and 14 which only had minor contributions. We narratively synthesised the evidence, focusing primarily on the strongest evidence.

## 3 Key evidence to inform policy

Policymakers can seek to empower staff through four main areas: 1) empowering through skills, 2) empowering through resources, 3) empowering through workforce structure, and 4) empowering through staff champions. See Fig. 1 for an overview of these areas.

## 4 Empowering through skills

The empowerment of health care staff to address the health needs of disadvantaged populations often involves targeted training. Key interventions typically target improving care for racial and ethnic minority groups, LGBTQ + people, and those with mental health diagnoses. Interventions include cultural competency, anti-racism, anti-stigma, advocacy, health literacy, and game-based training.

### 4.1 Cultural competency training

Cultural competence is the ability to understand values, attitudes, beliefs, and practices that differ across cultures, and respond appropriately to these differences. Systematic reviews consistently report that cultural competency training improves healthcare staff's knowledge, skills, and attitudes, though evidence linking training to improved patient outcomes is limited. Duckhee and colleagues reviewed 11 studies, including five randomized controlled trials, and found that nine showed positive effects on staff knowledge and attitudes, though only one study demonstrated improved patient satisfaction [10]. Most interventions used classroom-based learning and targeted specific healthcare professions, such as nurses or doctors, with limited follow-up to assess long-term effects.

In a mapping of 64 cultural competency interventions across the US, New Zealand, Canada, and Australia, Jongen and colleagues categorized them into two approaches: ‘categorical’ (focused on specific ethnic groups) and ‘cross-cultural’ (general skills for navigating cultural differences) [11]. Both approaches improved staff competencies, but lacked reporting of patient outcomes, with concerns that the categorical approach was prone to potential stereotyping.

McCann and colleagues assessed 44 studies on LGBTQ + competency training, finding improvements in knowledge, skills, and affirming practices [12]. However, while training increased awareness, its impact on healthcare workers’ biases and behaviours was limited, highlighting the challenges in translating knowledge into practice.

A review of 21 studies on training staff to deliver culturally adapted psychotherapies found that these approaches improved depressive symptoms, therapeutic alliance, and medication adherence among minority patients [13]. Successful interventions incorporated preparation, adaptation of therapies to cultural beliefs, and engagement with broader social systems.

### 4.2 Anti-racist and anti-stigma training

Anti-racist training aims to educate staff on systemic racism and equip them to challenge biases. Cénat and colleagues reviewed 30 studies and found that such training improved awareness of privilege, oppressive behaviours, and anti-racist practices. However, the studies lacked data on patient outcomes [14]. Similarly, Melro and colleagues highlighted the immediate benefits of addressing colonialism in health outcomes for indigenous people (e.g. improved knowledge, beliefs, attitudes, and behaviour), but noted the absence of long-term monitoring and patient-centred metrics [15].

Wong and colleagues reviewed 25 studies and found that educational interventions, including role-playing and games, effectively improved attitudes toward patients with mental illness [16]. Face-to-face interventions were more effective than online ones, suggesting that tailored, interactive formats enhance engagement.

### 4.3 Advocacy, literacy, and game-based training

Scott and colleagues categorized 78 studies on advocacy training for medical trainees into four intervention types, though did not assess effectiveness: classroom-based, clinical placements, field observations, and hybrid models [17]. Advocacy in healthcare contexts comprises supporting patients to express their views or wishes when communicating with healthcare workers. Commonly described concepts in the interventions included adapting practice to patient needs, social accountability, and mobilizing resources.

Several studies have found that training healthcare staff to support patients with low health literacy helps to improve understanding and engagement [18,19]. Successful interventions included integrated knowledge acquisition and skill development within real-world settings.

Game-based interventions, using electronic or board games, have also demonstrated positive effects on staff engagement with health equity concepts. Allan and colleagues reviewed 13 studies and found that game-based approaches enhanced learning, though again patient outcome data were lacking [20].

## 5 Empowering through resources

Effective empowerment of healthcare staff requires adequate time, culturally competent resources, and innovative care models such as trauma-informed care (TIC).

### 5.1 Adequate time

Time constraints pose significant barriers to delivering equity-focused care. Brown and colleagues reported that, while an intervention to improve attendance at speech and language services for low-income families was successful, competing job priorities limited proactive follow-up [21]. In primary care, GPs addressing social issues required longer consultations, yet evidence suggests that GPs in deprived areas have shorter appointments (even adjusting for health problems), exacerbating disparities [22].

### 5.2 Culturally competent resources

Providing culturally appropriate resources enhances staff capacity to address health inequities. A USA-based quality improvement project demonstrated improved mental health outcomes among Black and Latino adults when staff used culturally tailored resources [23]. However, evidence from the UK suggests that translating resources alone can create barriers, as formal language styles may hinder comprehension [24].

### 5.3 Trauma-informed care (TIC)

TIC emphasises understanding trauma's impact on health and considers how healthcare practices and environments can be set up to avoid re-traumatisation. Brown and colleagues reviewed 10 studies on TIC in emergency departments, identifying education, collaboration, and safety as critical themes [25]. Educational components improved staff awareness, while collaborative efforts with community organisations supported holistic care. Ensuring safety for patients and staff was central to intervention success.

## 6 Empowering through workforce structures

Creating an equitable workforce requires diversifying staff representation, enhancing professional development, and retaining employees from underrepresented backgrounds.

### 6.1 Increasing workforce diversity

Efforts to diversify the workforce are limited but promising. The NHS Cadet Scheme, though decommissioned, increased participation from Black and Asian backgrounds [26]. Mentorship programs in nursing education have proven effective in improving grades and admissions for underrepresented students, particularly when mentors share cultural backgrounds [27,28]. In the UK, outreach events in schools and the community to encourage people from minority groups to study nursing or midwifery increased knowledge and positive attitudes among south Asian participants [29]. In surgical training, longitudinal mentoring has been shown to increase racial minority participation [30]. In a study of hospitals as anchor institutions, Gkiouleka and colleagues found that in-person events such as job fairs and open days, and simplification of application forms supported people from disadvantaged backgrounds into work [31].

### 6.2 Increasing diversity in senior positions

Barriers to talent management (the recruitment, retention, and progression of staff to senior roles) disproportionately affect women and minority staff. Powell and colleagues highlighted challenges in NHS talent schemes, emphasizing the need for inclusive support [32]. A review of 62 studies examining talent management in nursing underscored mentorship's role in career progression for internationally recruited nurses, noting that cultural integration and communication are essential for job satisfaction [33].

### 6.3 Retaining staff from underserved communities

Retention strategies include fostering cultural safety, teamwork, supervision, professional development, and recognition. Deroy and colleagues found that cultural safety—where non-Aboriginal staff demonstrate culturally sensitive practices—was pivotal for retaining Aboriginal health staff in Australia [34].

## 7 Empowering through staff roles

Designating staff as champions for health inequalities can promote equity-focused care. While we did not find any quantitative evidence, qualitative findings highlight their role in fostering collaboration and innovation.

### 7.1 Health inequalities champions

Champions can drive systemic change and support staff in delivering equitable care. Coverdale and colleagues found that homeless care champions facilitated partnerships and improved end-of-life care [35]. Similarly, Wood and colleagues noted that clinical champions enhanced staff engagement and implementation of evidence-based practices in drug and alcohol and mental health settings [36]. A review of evidence about champions reported that five of the seven included studies found champions supported adoption of best practice in technological innovations [37].

## 8 Further considerations and implications

### 8.1 Limitations of the literature

Much of the literature focuses on high-income countries, limiting the generalisability of findings to low- and middle-income settings. There is a lack of longitudinal studies evaluating the long-term impact of interventions, which hinders the ability to assess sustainability. Most of the reviews included studies that were relatively small scale and tended to focus on staff outcomes rather than patient outcomes, meaning our understanding is limited of whether patients received or recognised the benefits of staff interventions. Linked to this, workforce interventions can be challenging to evaluate, given the complex contexts in which they are implemented.

### 8.2 Recommendations for policy

Health care organisations should ensure training for staff that covers.

• Cultural competency training, where appropriate focused on minoritised ethnic groups, LGBTQ + issues, or mental health, because it improves knowledge, skills, and attitudes of staff
• Anti-stigma mental health and anti-racist training
• Training to understand the perspective of people with low health literacy
• Advocacy

Health care organisations should ensure that staff have enough time to care for patients facing complex social circumstances and to undertake equity-focused service improvement projects. Relatedly, further research is needed that evaluates the impacts of such interventions on patient outcomes. Furthermore, staff should have access to culturally competent resources and resources accessible to those with poor health literacy. Improving staff diversity, particularly at senior levels, may support these goals. Finally, trauma-informed care approaches should be promoted among staff, especially those in emergency departments and general practice.

## 9 Conclusion

Leveraging the health care workforce has significant potential to address health and care inequalities through staff training, ensuring adequate time and resource, increasing the diversity of the workforce and staff champion models. However, policy makers face challenges in implementing such solutions in the context of health care organisations facing competing pressure for resources.

## Ethics statement

Not applicable as this study is based on a literature review of existing research.

## Availability of data and materials

The datasets are available in the cited references.

## Authors' contributions

JF, DL and SH designed the study. DL and SH undertook the data extraction and synthesis. ADL, CG, HP, HPe, and HPa supported interpretation of the findings. DL led manuscript writing. All authors contributed to the final manuscript.

## Funding

This study was commissioned by 10.13039/100030827NHS England. This report is independent research supported by the National Institute for Health and Care Research ARC North Thames and NHS England. The views expressed in this publication are those of the author(s) and not necessarily those of the National Institute for Health and Care Research, NHS England or the Department of Health and Social Care.

## Declaration of competing interest

The authors declare the following financial interests/personal relationships which may be considered as potential competing interests:This report is independent research supported by the National Institute for Health and Care Research ARC North Thames and NHS England. The views expressed in this publication are those of the author(s) and not necessarily those of the National Institute for Health and Care Research, NHS England, or the Department of Health and Social Care.
